# A new type of ultrasonic water alleviates constipation with favorable safety

**DOI:** 10.3389/ftox.2025.1679872

**Published:** 2025-11-20

**Authors:** Jiarong Hu, Xinyi Hu, Wenjie Huang, Fen Wang, Zhongqi Xia, Chenyang Zhu, Junwei Chow, Shiwei Yan, Longzhou Li, Haiyang Liu, Shufen Cui, Guo Ma

**Affiliations:** 1 School of Pharmaceutical Sciences, State Key Laboratory of Advanced Drug Formulations for Overcoming Delivery Barriers, Fudan University, Shanghai, China; 2 Department of Pharmacy, Huashan Hospital Affiliated to Fudan University, Shanghai, China; 3 Department of Pharmacy, Affiliated Hospital of Shandong University of Traditional Chinese Medicine, Jinan, China

**Keywords:** ultrasonic water, constipation, purgative effect, safety, toxicity test

## Abstract

**Introduction:**

Ultrasonic water (UW) is a novel category of functional drinking water. This study aimed to investigate its laxative efficacy and underlying mechanisms using a constipation model in SD rats, while also conducting a comprehensive safety evaluation.

**Methods:**

A rat constipation model was established to assess the purgative effect of UW. Safety was systematically evaluated through acute oral toxicity tests, subacute oral toxicity tests, a bone marrow cell micronucleus test, and an Ames test. Serum levels of gastrointestinal hormones—including motilin, substance P, vasoactive intestinal peptide, acetylcholinesterase, and somatostatin—were measured to explore potential mechanisms.

**Results and discussion:**

UW significantly improved constipation symptoms of the test SD rats, with a significant increase in body weight (16.03 ± 5.32%) compared to the model group (8.67 ± 4.02%, P < 0.05). Compared to the model group, serum levels of excitatory gastrointestinal hormones were significantly elevated 4.20% (motilin), 10.87% (substance P), 36.69% (vasoactive intestinal peptide) and 12.89% (acetylcholinesterase) (P < 0.05), while somatostatin, was markedly reduced 22.40% (P < 0.05), respectively. Acute oral LD50 values of UW were higher than 20000 mg/kg, indicating practical non-toxicity. In the subacute toxicity test, compared to those in the control group, performances, blood routine indexes and serum biochemical indexes of the rats in the UW group showed no statistical difference (P > 0.05), and all the organs and tissues of the rats in both groups maintained normal morphology. Micronucleus effect of UW was not found when the dosage of UW was up to 20000 mg/kg. UW also showed no mutagenic activity on standard test strains. In summary, UW exhibited favorable purgative effect and safety.

## Introduction

Constipation is most simplistically defined as unsatisfactory defecation resulting from infrequent stools, difficult stool passage, or both (Scott et al., 2021). The worldwide incidence of constipation is approximately 15%–20%, and it has become a major healthcare burden (Sharma et al., 2021). The pathogenesis of constipation is complex. Different types of constipation (i.e., primary and secondary constipation) have different pathogenesis. Primary constipation is a consequence of neuromuscular dysfunction of the colon or anorectal sensory–motor function, while secondary constipation is associated with organic disease (e.g., colonic stricture, mass, or malignancy), medication use (e.g., opioids or anti-cholinergic medications), or an underlying condition (e.g., metabolic, thyroid, or diabetic disorders) (Sharma et al., 2021). Constipation can reduce the frequency of defecation, lead to dry stool and difficult defecation, and be accompanied by symptoms such as abdominal pain, loss of appetite, fatigue, and weakness, which affect people’s health and quality of life. Lifestyle modifications, a well-designed diet (e.g., increasing intake of fiber and water), and the administration of drugs with purgative effects are usually used to prevent and treat constipation, especially chronic constipation (Bellini et al., 2021).

**FIGURE 1:**
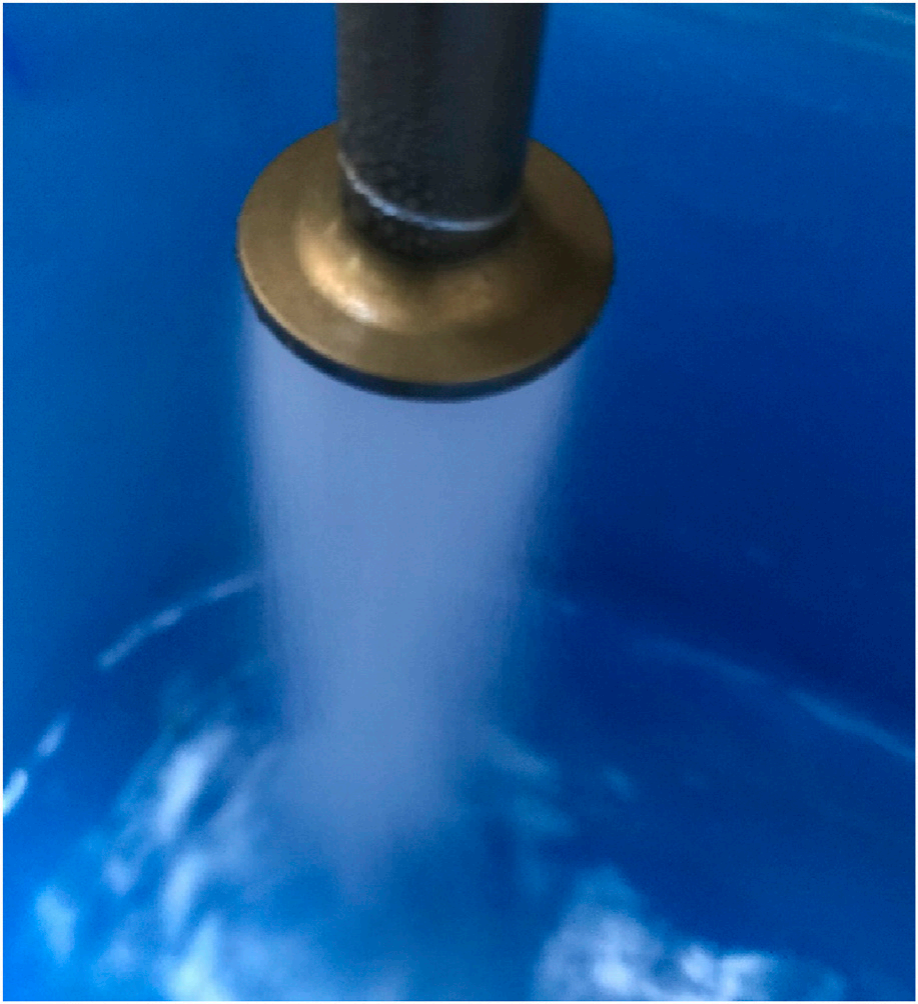
Ultrasonic water produced using ultrasonic water equipment.

Ultrasonic water (UW) (Figure 1) is a new type of functional drinking water produced by ultrasonic technology. UW is produced by cavitation, atomization, cutting, and energy gathering of water flow, leading to high temperature (more than 5,000 K), high pressure (5.05 × 10^7^ Pa), and a high rate of temperature changes (10^9^ K/s) using the ultrasonic technology, accompanied by strong shock waves and microjets (400 km/h). The water molecules rearrange and combine to form a cluster network structure n × (H_2_O)_n_ via the heat expansion and cold contraction effect of air–oxygen exchange. In addition, the nuclear magnetic resonance (NMR) experiment shows that the movement speed of water molecules in UW is 20% faster than that in ordinary water (Xia, 2019). After the treatment, UW displays better quality and a favorable taste and meets the quality criteria for drinking water. In particular, the disinfection by-products (e.g., trichloromethane and carbon tetrachloride) in domestic drinking water are removed through ultrasonic and purification treatment.

Interestingly, it was found that UW exhibits purgative function during long-term drinking, which can improve the constipation symptoms of the constipation crowd. However, until now, the purgative effect of UW has not been verified through rigorous studies, its purgative mechanisms have not been explained, and its safety has not been evaluated. In the present study, a constipation model was established in SD rats to study the purgative function, mechanisms, and safety of UW, thereby providing scientific evidence to support its application in a large population.

## Instruments and materials

### Instruments

The following instruments were used in this study: CS3058 domestic ultrasonic water equipment (Wuxi Tianxia Ultrasonic Equipment Co., Ltd., Wuxi, China), full-automatic biochemical analyzer (Hitachi 7170, Tokyo, Japan), five-classification blood cell counting instrument (Mindray BC-5380, Shenzhen, China), enzymatic marker (Thermo Fisher Scientific, MULTISKAN GO system), metabolic cage (Feng’s Laboratory Animal Equipment Co., Ltd.), biochemical incubator (Thermo Fisher Scientific, Waltham, MA, United States), centrifuge (Thermo Fisher Scientific), electronic balance (Saiduolisi BSA822, Shenzhen, China), rat fixator (Shanghai Huake Experiment Equipment Co., Ltd., Shanghai, China), dehydrator (DIAPATH Donatello, Martinengo, Italy), embedding machine (Wuhan Junjie Electronics Co., Ltd., JB-P5, Wuhan, China), pathology slicer (Leica RM 2016, Wetzlar, Germany), frozen platform (Wuhan Junjie Electronics Co., Ltd., JB-L5), organizer (KEDEE KD-P, Taizhou, China), dyeing machine (DIAPATH Giotto), oven (Laboratory GFL-230), glass slide (Servicebio G6004, Wuhan, China), upright optical microscope (Nikon Eclipse ci), and imaging system (Nikon DS-F12).

### Reagents

The following reagents were used in this study: UW (UW was produced using CS3058 domestic ultrasonic water equipment, and its parameters were as follows: pH 8.0, conductivity of 1–5 μS/cm (25 °C), heavy metals far below the international standards, and no detectable chemical residues); purified water (Nestlé, 20220226); Tongbianling (TBL) capsule (batch number 20220302, Jinan Limeng Pharmaceutical Co., Ltd.); compound diphenoxylate tablets (batch number 2105014, Jiangsu Changzhou KangPu Pharmaceutical Co., Ltd., China); rat motilin (MTL), substance P (SP), vasoactive intestinal peptide (VIP), gastrin (GAS), acetylcholinesterase (AChE), endothelin (ET), and somatostatin (SS) ELISA test kits (batch number 202012, Shanghai Hengyuan Biotechnology Co., Ltd.); serum alanine aminotransferase (ALT), aspartate aminotransferase (AST), total protein (TP), albumin (ALB), globulin (GLO), total bilirubin (TBIL), uric acid (UA), blood urea nitrogen (BUN), creatinine (CRE), cholesterol (CHO), triacylglycerol (TG), and blood glucose (GLU) test reagents (Guangzhou Kehua Biotechnology Co., Ltd.); M-53LH hemolytic agent, M-53LEO (I) hemolytic agent, M-53LEO (II) hemolytic agent, M-53 detergent, and diluent (Shenzhen Mindray Biomedical Electronic Co., Ltd.); ethanol (batch number 100092683, SCRC); xylene (batch number 10023418, SCRC); HE dye solution set (batch number G1003, Servicebio); neutral gum (batch number 10004160, SCRC); cyclophosphamide (Sigma-Aldrich, Merck); S9 (rat liver homogenate induced by β-naphthoflavone and phenobarbital sodium, CHI SCIENTIFIC); sodium azide (Sigma-Aldrich, Merck); Dexon (Sigma-Aldrich, Merck); 2-aminofluorene (2-AF, Sigma-Aldrich, Merck); 1,8-dihydroxyanthraquinone (1,8-DHAQ, Sigma-Aldrich, Merck); DMSO (Sigma-Aldrich, Merck); and *S. typhimurium* strains TA97a, TA98, TA100, and TA102.

### Animals

The SD rats (SPF grade, 200 ± 20 g, n = 72, half male and half female, provided by the Experimental Animal Center, School of Pharmaceutical Sciences, Fudan University) and the ICR mice (SPF grade, 20 ± 2 g, n = 70, half male and half female, provided by the Experimental Animal Center, School of Pharmaceutical Sciences, Fudan University) were used in this study. The animal production license number was SCXK(Hu)2018-0006. The temperature was set at 25 °C ± 2 °C, and the humidity was kept at 45%–60%. All the animal care and experimental protocols complied with the Animal Management Rules of the Ministry of Health of the People’s Republic of China and were approved by the Animal Ethics Committee of the School of Pharmaceutical Sciences, Fudan University (ethics approval no. 2022-03-LY-MG-43, Shanghai, China).

## Methods

### Purgative function of UW

#### Preparation of the solution

Compound diphenoxylate tablets (each tablet containing 2.5 mg of diphenoxylate hydrochloride and 0.025 mg of atropine sulfate) were ground into superfine powders using a mortar, and the compound diphenoxylate suspension (5 mg/mL) was prepared with purified water. TBL suspension (50 mg/mL) was prepared by suspending the contents of TBL capsules (a commonly used drug for treating constipation) in purified water.

#### Construction of the SD rats with constipation

The SD rats with constipation were established according to the studies by Xie and Zhou (2014), Peng et al. (2007), Wu et al. (2011), Liu et al. (2012), and Xin et al. (2014). The rats were fasted (with free access to water) for 12 h and were randomly divided into two groups. One group (8 rats, the normal group) was intragastrically administered an equal amount of purified water for 9 days and served as the control group. The other group (24 rats, the model group) was intragastrically administered a compound diphenoxylate suspension (25 mg/kg) for 9 days, successfully establishing a constipation model in SD rats.

#### Animals grouping

Except for the normal group, the 24 SD rats with constipation (the model group) were randomly divided into three groups (8 rats per group, half male and half female), i.e., the model group, the positive group, and the UW group. Each rat in the normal and model groups was intragastrically administered a quantitative amount of purified water (200 mL/kg body weight) every day. Each rat in the positive group was intragastrically administered a TBL suspension (250 mg/kg) and simultaneously given a quantitative amount of purified water (195 mL/kg body weight) every day. The UW group was intragastrically administered ultrasonic water (200 mL/kg body weight).

#### General behavior observation

Daily behaviors (i.e., mental state, activity, feed and water intake, fur, and defecation) of the SD rats in the normal, model, positive, and UW groups were observed. The rats were weighed every 2 days.

#### Detection of the serum biochemical indices

The rat blood was taken at three time points (before the compound diphenoxylate suspensions were given, after the rat constipation models were successfully established, and at the end of the whole experiment). The blood was then centrifuged at 3,000 r/min for 5 min, and the obtained serum was used to detect the biochemical indices related to constipation, i.e., MTL, SP, VIP, GAS, AChE, ET, and SS.

### Safety evaluation of UW

#### Acute oral toxicity test

After being fasted overnight, a total of 20 ICR mice, half male and half female, were intragastrically administered 20 mL/kg of UW. Four hours later, the mice were given water and feed, and their general behavior, toxic reactions, and toxic symptoms were observed for 14 days.

#### Subacute oral toxicity test

The SD rats (40 rats) were randomly divided into two groups, i.e., the control group and the UW group (20 rats per group, half male and half female), which were given purified water and UW, respectively. The subacute oral toxicity test, including the general behavior observation, blood routine examination, serum biochemical index determination, and histopathological examination, was conducted to evaluate the safety of UW.

1. General behavior observation: The behaviors of the SD rats in the control and UW groups were observed and recorded daily for four consecutive weeks. The rats were weighed once a week. The feed and water intake of the rats were recorded twice a week. The weekly intake of feed and water was calculated.
2. Blood routine examination: The blood was taken from the ocular venous plexus of the SD rats at weeks 0, 2, and 4. The blood routine indices [i.e., white blood cell (WBC), lymphocyte (LYMPH), monocyte (MONO), neutrophil (NEUT), eosinophil (EO), basophil (BASO), red blood cell (RBC), hemoglobin (HGB), hematocrit (HCT), mean corpuscular volume (MCV), mean corpuscular hemoglobin (MCH), mean corpuscular hemoglobin concentration (MCHC), red blood cell distribution width-standard deviation (RDW-SD), red blood cell distribution width-coefficient of variation (RDW-CV), platelets (PLT), mean platelet volume (MPV), plateletcrit (PCT), and platelet distribution width (PDW)] were determined.
3. Serum biochemical index determination: According to the method “blood routine examination,” the serum biochemical indices of the SD rats [i.e., ALT, AST, ALT/AST, TP, ALB, GLO, ALB/GLO (A/G), TBIL, UA, BUN, CRE, CHO, TG, and GLU] were determined.
4. Histopathological examination: After 4 weeks, rats were fasted overnight with free access to water before dissection. Anesthesia was induced by inhalation of isoflurane (2% concentration, delivered at a fresh gas flow rate of 4 L/min with 0.41 mL/min liquid isoflurane vaporization), followed by blood collection via transabdominal aortic puncture. Then, the heart, liver, spleen, lungs, kidneys, brain, stomach, duodenum, jejunum, ileum, colon, cecum, rectum, uterus, ovaries, testes, and skeletal muscles of the rats were collected. The collected tissue samples were fixed, dehydrated through a graded series of alcohols and xylene, embedded in paraffin, and sectioned at 4 μm. Subsequently, the sections were stained with hematoxylin and eosin (H&E) following standard protocols and examined under a light microscope for morphological analysis.

#### Micronucleus test of bone marrow cells

The ICR mice (50 mice) were randomly divided into three groups (half male and half female for each group), i.e., the negative control group (10 mice), the positive control group (10 mice), and the UW group (30 mice in total, 10 mice each in low-, middle-, and high-dose groups), which were intragastrically administered purified water, cyclophosphamide, and UW, respectively. The low, middle, and high doses of administered UW were 5,000, 10,000, and 20,000 mg/kg (equivalent to 5, 10, and 20 mL/kg of UW), respectively, in the UW group. Then, 5 and 10 mL of UW were diluted to 20 mL with purified water. The ICR mice in the negative control group (purified water), the positive control group (cyclophosphamide, 40 mg/kg), and the UW group (low-, middle-, and high-dose UW) were all intragastrically administered 20 mL/kg twice within 30 h. The mice were euthanized by inhalation of isoflurane (2% concentration, delivered at a fresh gas flow rate of 4 L/min with 0.41 mL/min liquid isoflurane vaporization) 6 h after the second gavage, and the bone marrow cell suspension was prepared with calf serum for smears. The micronucleus rate and the ratio of polychromatic erythrocytes (PCEs) to normochromic erythrocytes (NCEs) were calculated.

#### Ames test

Four dose groups of UW, i.e., 12.5, 25, 50, and 100 μL/dish, were set. The positive control strains were TA97a, TA98, and TA102 with Dexon (50.0 μg/dish) and TA100 with NaN_3_ (1.5 μg/dish) in the absence of S9; TA97a, TA98, and TA100 with 2-AF (10.0 μg/dish) and TA102 with 1,8-DHAQ (50.0 μg/dish) in the presence of S9. The positive control substances, NaN_3_, Dexon, 2-AF, and 1,8-DHAQ, were prepared with sterilized double-distilled water or sterilized DMSO. The solvent and blank control groups (spontaneous revertants) were also set. The experiments were performed using the flat mixing method. Three parallel dishes are set for each dose group. A volume of 0.1 mL of the test strain enrichment solution and 0.1 mL of UW were added to the top agar. A volume of 0.5 mL of S9 mixture was added to the top agar for metabolic activation. After they were mixed, the mixture was poured onto the bottom medium plate. After the medium solidified, it was placed upside down and incubated at 37 °C for 48 h. The number of revertant colonies per dish in each test group was recorded. When the number of revertant colonies in the UW group is at least twice that of the solvent control value and shows a dose-response relationship, it is considered mutagenic positive.

### Statistical analysis

All the experimental data were analyzed using statistical analysis software SPSS 20.0 and were recorded as the means ± standard deviations. Analysis of variance or a chi-square test was used to compare differences among the multiple groups, and the LSD-t test was used to compare the differences between two groups. *p < 0.05* indicates a statistically significant difference.

## Results

### Purgative effect of UW

#### General behavior

After the compound diphenoxylate suspensions were administered, the SD rats with constipation in the model group showed symptoms of poor mental state, clumping, reduced activity, reduced food intake, abdominal distension, hair standing on end and shedding, fewer defecation times and volumes, smaller stool size, bulbous stool, and dry, hard stool. After the rats were administered purified water, TBL suspension, and UW, respectively, the above-mentioned symptoms in the SD rats in the positive and UW groups were significantly improved, showing more defecation times and volume, increased food intake, increased activity, and wetter stool. This indicated that UW had a defecation function and could improve constipation symptoms.

#### Body weight changes

**FIGURE 2:**
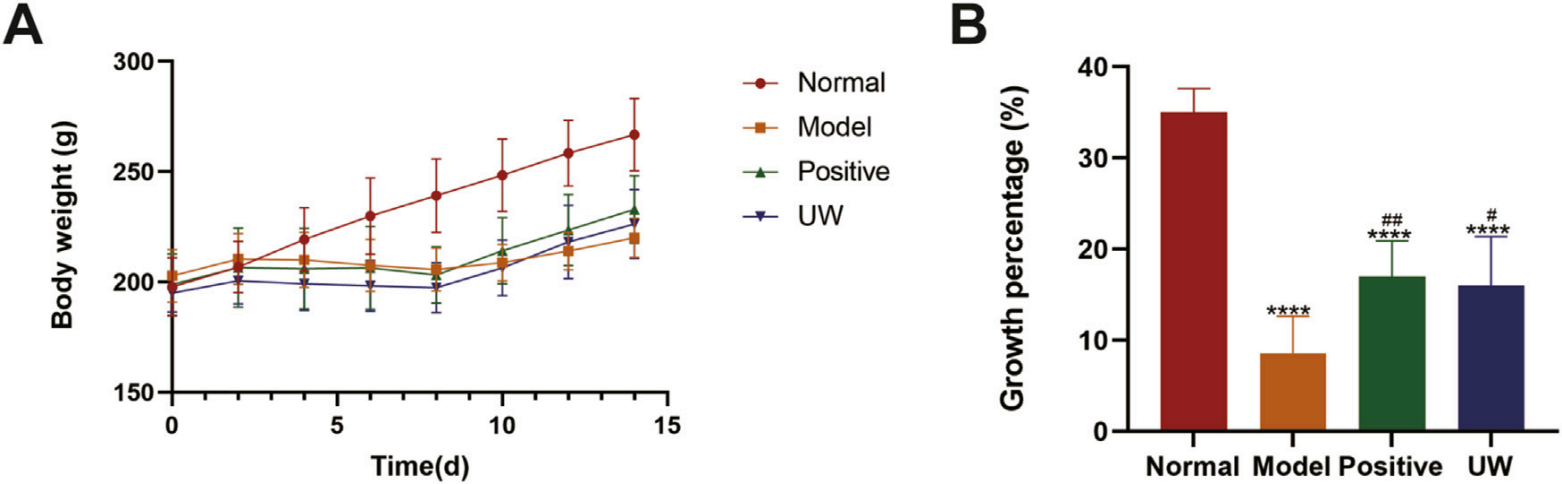
Change trend and growth percentage of body weight of the SD rats (n = 8). Note: **(A)** the change trend of body weight in the SD rats; **(B)** the growth percentage of body weight in the SD rats. Normal represents the normal SD rats; Model represents the SD rats with constipation; Positive represents the positive control SD rats administered TBL suspension (250 mg/kg); UW represents the SD rats given ultrasonic water. Compared to the normal group, ∗∗∗∗, *p <* 0.0001; compared to the model group, #, *p <* 0.05, and ##, *p <* 0.01.

Compared to that of the normal group, the body weight of the rats with constipation decreased. This indicated that constipation can reduce the body weight of the rats. After 14 days, the weight growth rates of the rats in the normal, model, positive, and UW groups were (34.92 ± 2.59) %, (8.67 ± 4.02) %, (17.16 ± 3.99) %, and (16.03 ± 5.32) %, respectively. Compared to that of the rats in the model group, body weight growth rates of the rats in the positive and UW groups significantly increased (*p < 0.05*) (Figure 2). This indicated that both TBL and UW improved the body weight of the rats with constipation. It should be noted that the body weight and its growth rates of the rats in the model, positive, and UW groups were lower than those of the rats in the normal group. This implied that TBL and UW only improved the body weight of rats with constipation to a certain extent, which was due to the short experimental period (i.e., 14 days).

#### Serum biochemical index

**FIGURE 3:**
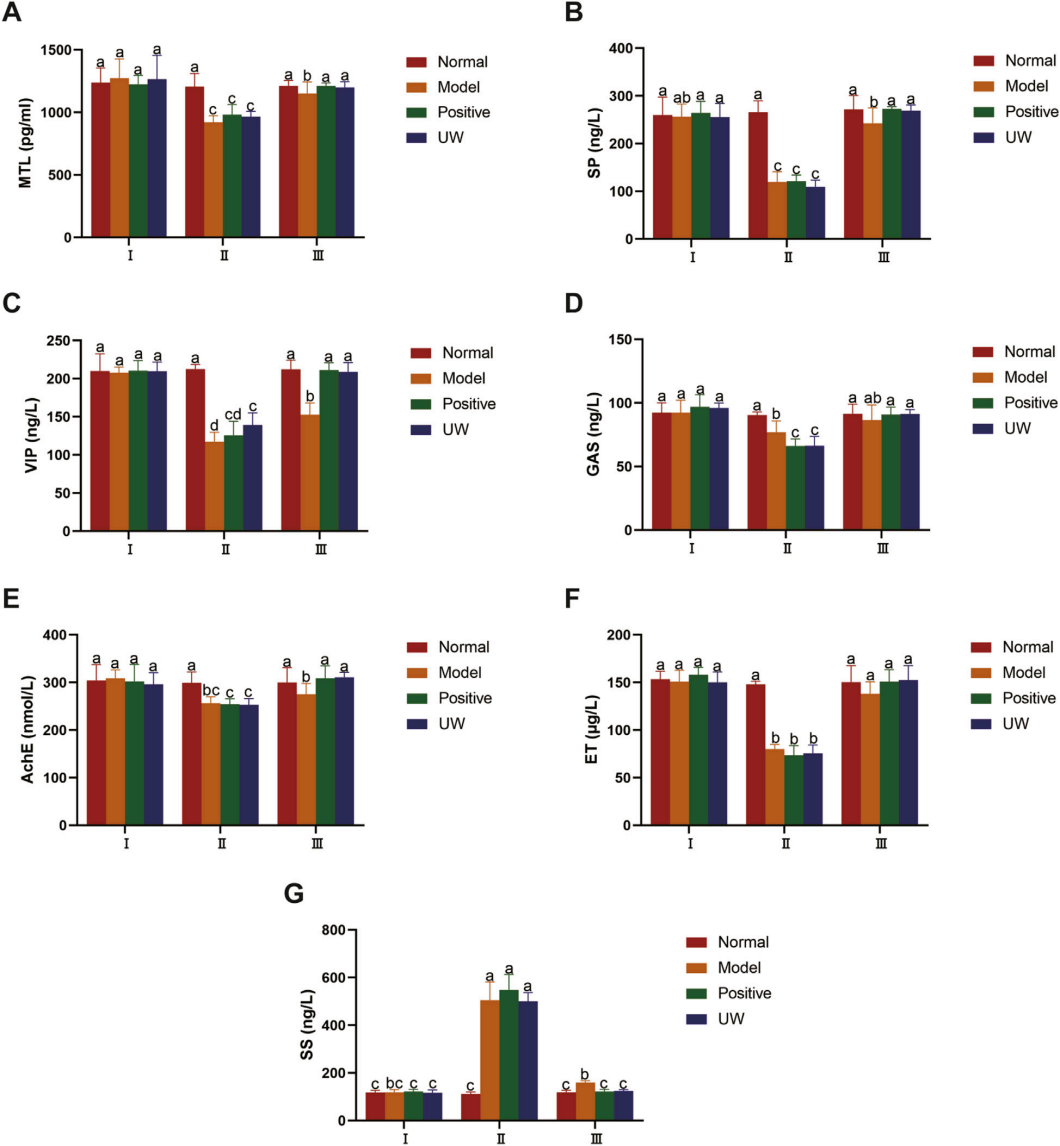
Biochemical indexes related to defecation (MTL, SP, VIP, GAS, AchE, ET, SS) of the SD rats (n = 8). Note: **(A)**, Motilin (MTL); **(B)**, Substance P (SP); **(C)**, Vasoactive intestinal peptide (VIP); **(D)**, Gastrin (GAS); **(E)**, Acetylcholinesterase (AChE); **(F)**, Endothelin (ET); **(G)**, Somatostatin (SS). I, before the compound diphenoxylate suspensions were given the rats (Day 0); II, when the rat constipation models were successfully established (Day 9); III, at the end of the whole experiment (Day 23). Normal represents the normal SD rats; Model represents the SD rats with constipation; Positive represents the positive control SD rats administrated TBL suspension (250 mg/kg); UW represents the SD rats given ultrasonic water. a, b, and c, the mean values with different letters over the bars are significantly different (P < 0.05), the mean values with the same letters over the bars show no significantly difference (P > 0.05).

Before the compound diphenoxylate suspensions were administered (Day 0, I), the biochemical indices (i.e., the indicators related to constipation MTL, SP, VIP, GAS, AChE, ET, and SS) in the rats among the normal, model, positive, and UW groups showed no significant difference *(p > 0.05*). However, after the compound diphenoxylate suspensions were administered for 9 days (Day 9, II), compared to those in the rats in the normal group (without the compound diphenoxylate suspensions), serum levels of MTL, SP, VIP, GAS, AChE, and ET decreased (*p < 0.05*), while the serum level of SS increased (*p< 0.05*) in the model, positive, and UW groups. This indicated that constipation models in the rats were successfully established in the model, positive, and UW groups, which was consistent with a previous report (Liu et al., 2019). After the rats were administered purified water in the normal and model groups, TBL suspension in the positive group, and UW in the experimental group for 14 days (Day 23, III), the serum levels of MTL, SP, VIP, AChE in the rats significantly increased (*p < 0.05*), while the serum level of SS significantly decreased (*p < 0.05*) in the positive and UW groups compared with those in the rats in the model group. Moreover, compared to those of itself (I) or the normal group (III), all the biochemical indices in the rats in the positive and UW groups showed no significant difference (*p > 0.05*), suggesting that these biochemical indices recovered the normal level after the rats with constipation were administered UW and TBL. All these indicated that both UW and TBL exhibited a favorable defecation effect. In summary, all the biochemical indices showed no statistically significant differences between the positive and UW groups (*p > 0.05*), indicating that UW had the same defecation effect as TBL. The detailed biochemical indices of the rats in the normal, model, positive, and UW groups are shown in Figure 3 and Supplementary Table S1.

### Safety of UW

#### Acute oral toxicity test

No obvious signs of poisoning were observed in male or female mice administered UW within 2 weeks. No animal died during the observation period, and no significant abnormalities were found in gross anatomy. The result indicated that the oral LD_50_ values of UW in the male and female mice were more than 20,000 mg/kg, and UW was practically non-toxic (Table 1).

**TABLE 1:** Death number of the mice and LD_50_ value.

Dose (mg/kg b.wt.)	Death number/Experiment number		LD_50_ (mg/kg b.wt.)	
	Male	Female	Male	Female
20,000	0/10	0/10	>20,000	>20,000

b. wt, body weight.

#### Subacute oral toxicity test

**FIGURE 4:**
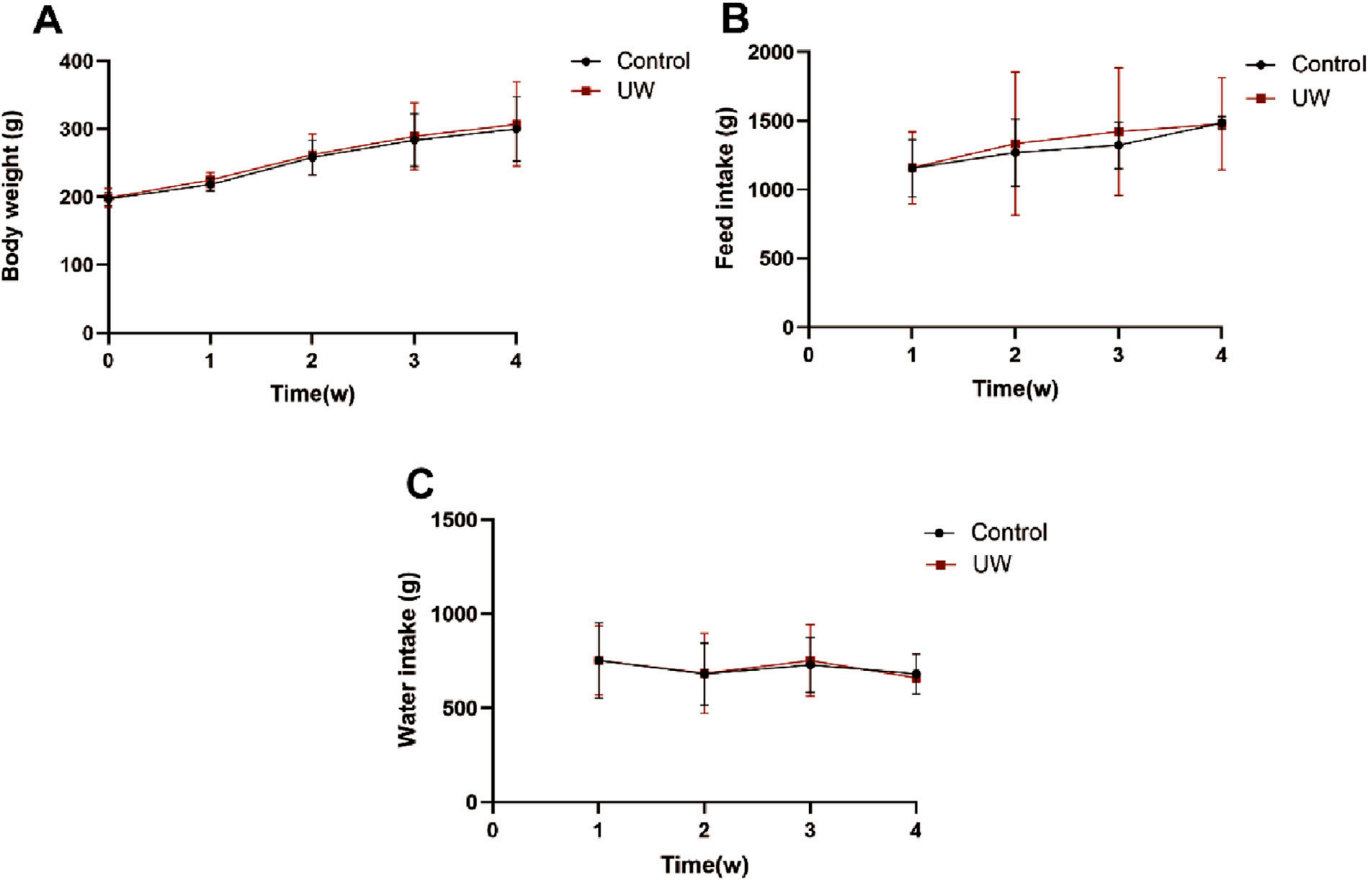
Change trend in the body weight **(A)**, feed intake **(B)**, and water intake **(C)** of the SD rats during the 4 weeks. Note: The values are expressed as the means ± SD of 10 rats in each group.

**FIGURE 5:**
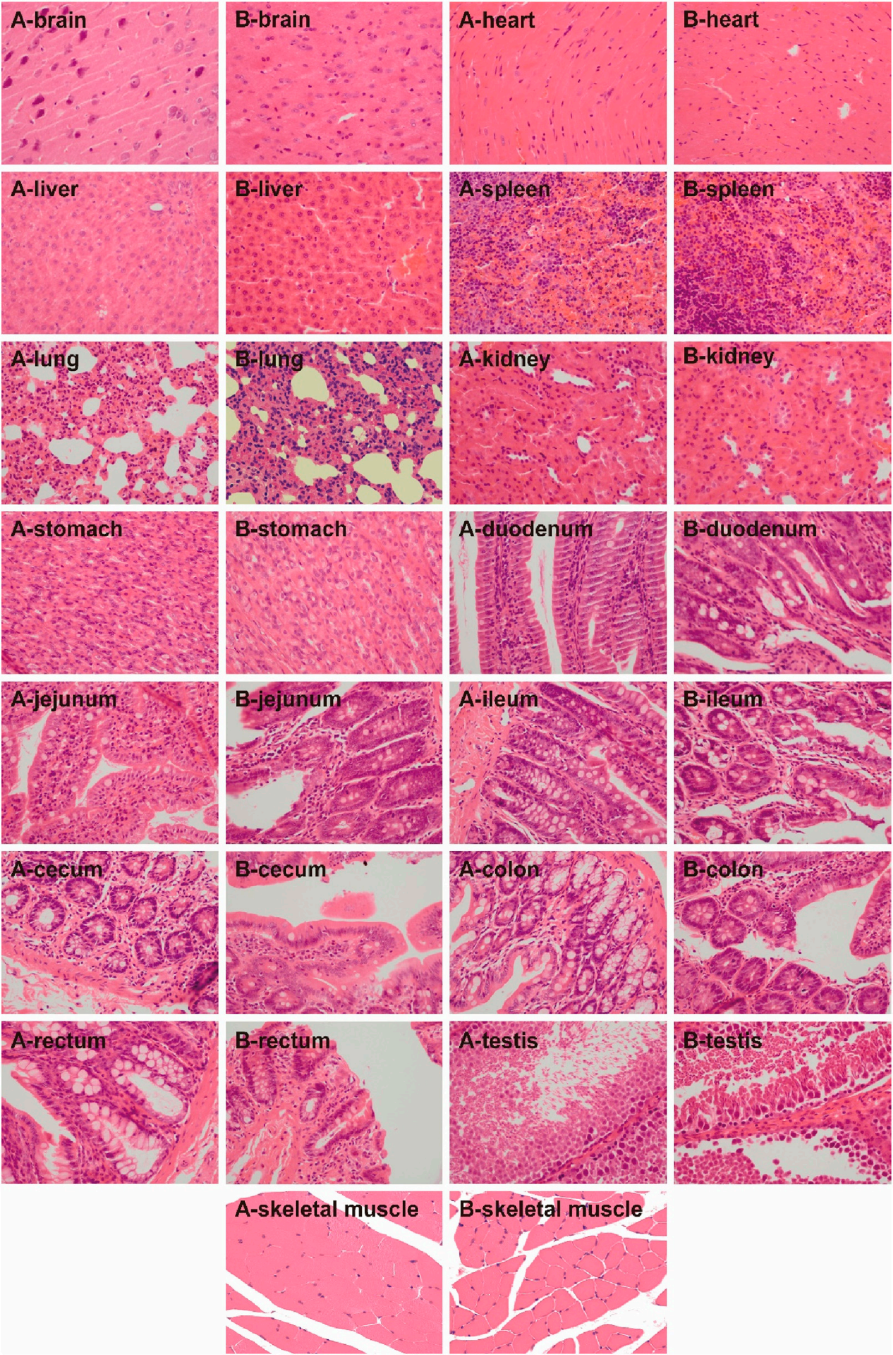
The H&E-stained images of the tissues of the male rats in the control and UW groups, respectively (×400). Note: A represents the control group, and B represents the UW group.

**FIGURE 6:**
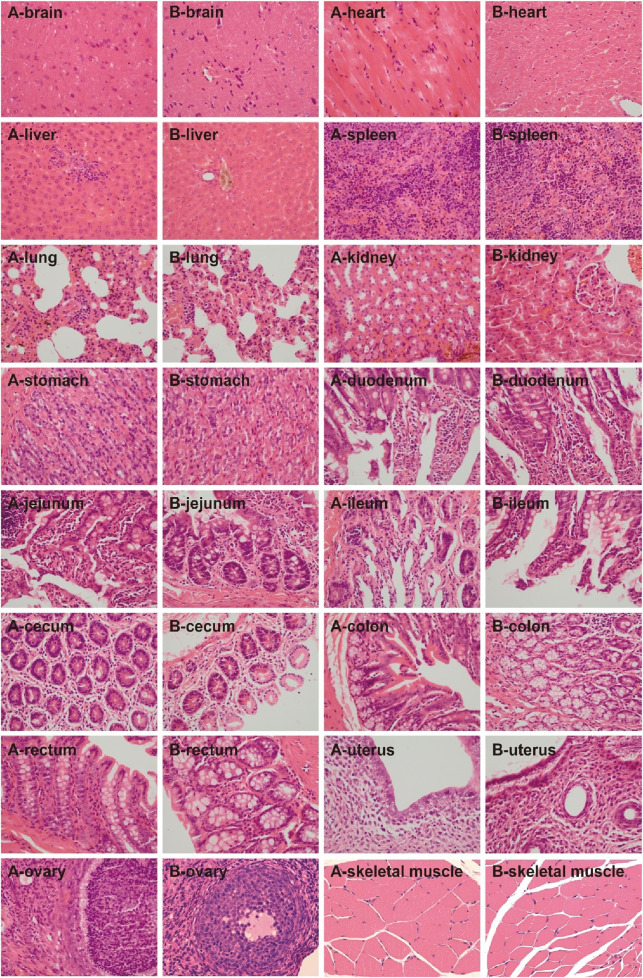
The H&E-stained images of the tissues of the female rats in the control and UW groups, respectively (×400). Note: A represents the control group, and B represents the UW group.

1. General behavior: During the 4-week administration, no food refusal was observed in the UW and control groups. The body weight, feed intake, and water intake in the UW group showed no significant differences compared to those in the control group (*p > 0.05*) (Figure 4).
2. Serum biochemical indices: Serum biochemical indices in the SD rats in the control and UW groups at weeks 0, 2, and 4 of the experiment are shown in Table 2. Compared with that in the control group, the UA level of the rats in the UW group significantly decreased (*p < 0.05*) in the second week. The AST and total protein levels of the rats in the UW group significantly increased (*p < 0.05*) in the fourth week. The other serum biochemical indices of the rats showed no statistically significant differences (*p > 0.05*) between the control and UW groups. It should be noted that, although some serum biochemical indices (e.g., UA, AST, and total protein) of the rats between the UW and control groups showed statistically significant differences, these differences had no clinical significance because they were within the normal physiological range. In short, UW did not cause abnormality in the serum biochemical level and potential hepatorenal toxicity in rats.
3. Blood routine indices: Blood routine indices of the SD rats between the control and UW groups at weeks 0, 2, and 4 of the experiment are shown in Table 3. Except for the mean neutrophil counts (NEUT), all other blood routine indices of the SD rats showed no statistically significant differences (*p > 0.05*) between the control and UW groups at weeks 0, 2, and 4. Compared with that of the control group, NEUT of the SD rats in the UW group significantly decreased (*p < 0.05*) in the second week and showed no statistically significant differences (*p > 0.05*) in the fourth week. However, the difference in NEUT between the control and UW groups had no clinical significance because NEUT was within the normal physiological range. In addition, compared with those at week 0, the blood routine indices of the SD rats in the second and fourth weeks showed no statistically significant differences (*p > 0.05*). In brief, UW did not lead to significant changes in the blood routine indices of the SD rats.
4. Histopathological characteristics: In the histopathological experiment, all the organs and tissues of the male and female rats in the control and UW groups maintained normal histological structure and cell morphology and showed no significant pathological changes and toxicological characteristics (Figures 5, 6; Supplementary Table S2).

**TABLE 2:** Serum biochemical levels in the SD rats during the experimental period (*n* = 10).

Index (unit)	0 w		2 w		4 w	
	Control group	UW group	Control group	UW group	Control group	UW group
ALT (U/L)	80.80 ± 12.36	72.10 ± 9.18	83.10 ± 6.74	83.10 ± 6.95	63.00 ± 9.21	69.70 ± 13.46
AST (U/L)	179.80 ± 53.01	143.30 ± 27.86	175.10 ± 52.23	158.10 ± 35.62	162.90 ± 25.69	200.80 ± 38.29^a^
AST/ALT	2.22 ± 0.52	2.03 ± 0.50	2.14 ± 0.70	1.79 ± 0.37	2.60 ± 0.28	2.92 ± 0.47
TP (g/L)	61.20 ± 5.98	60.00 ± 4.47	62.60 ± 5.50	64.30 ± 6.75	75.76 ± 3.63	80.66 ± 5.21^a^
ALB (g/L)	32.70 ± 2.16	33.70 ± 2.50	33.90 ± 2.81	35.00 ± 3.33	42.66 ± 9.78	47.76 ± 3.56
GLO (g/L)	28.40 ± 3.86	26.30 ± 2.11	28.70 ± 2.87	29.30 ± 4.08	33.10 ± 9.31	32.90 ± 2.33
A/G	1.16 ± 0.08	1.18 ± 0.07	1.18 ± 0.06	1.21 ± 0.14	1.40 ± 0.40	1.46 ± 0.12
TBIL (μmol/L)	0.43 ± 0.32	0.37 ± 0.35	0.58 ± 0.30	0.44 ± 0.29	0.85 ± 0.32	0.76 ± 0.47
UA (μmol/L)	113.80 ± 26.99	94.00 ± 14.51	115.70 ± 15.30	95.20 ± 18.6^a^	115.30 ± 16.47	123.50 ± 25.26
BUN (mmol/L)	5.28 ± 0.94	4.62 ± 0.62	6.28 ± 0.87	6.64 ± 1.35	7.78 ± 0.74	7.63 ± 1.29
CRE (μmol/L)	37.20 ± 14.87	26.70 ± 5.98	37.20 ± 8.23	31.40 ± 6.38	41.80 ± 7.38	47.30 ± 9.53
CHO (mmol/L)	2.09 ± 0.73	2.41 ± 0.31	2.20 ± 0.47	2.22 ± 0.33	2.13 ± 0.54	2.35 ± 0.35
TG (mmol/L)	0.68 ± 0.31	0.71 ± 0.22	1.01 ± 0.49	1.04 ± 0.43	0.97 ± 0.49	0.93 ± 0.36
GLU (mmol/L)	7.51 ± 0.98	7.70 ± 0.64	8.17 ± 1.07	8.94 ± 1.15	10.10 ± 1.21	9.20 ± 1.85

w, week; ALT, serum alanine aminotransferase; AST, aspartate aminotransferase; TP, total protein; ALB, albumin; GLO, globulin; TBIL, total bilirubin; UA, uric acid; BUN, urea nitrogen; CRE, creatinine; CHO, cholesterol; TG, triacylglycerol; GLU, blood glucose. The values are expressed as the means ± SD of 10 rats in each group. Compared to the control group, a, *p <* 0.05.

**TABLE 3:** Blood routine indices in the SD rats during the experimental period (n = 10).

Index (unit)	0 w		2 w		4 w	
	Control group	UW group	Control group	UW group	Control group	UW group
WBC (10^9^)	14.64 ± 2.64	12.38 ± 2.55	14.84 ± 1.88	13.12 ± 2.07	13.20 ± 2.82	12.36 ± 1.91
LYMPH (%)	68.24 ± 7.80	73.75 ± 4.22	73.80 ± 6.59	75.60 ± 3.87	72.94 ± 9.30	77.03 ± 5.48
MONO (%)	1.39 ± 0.93	1.25 ± 1.05	1.26 ± 0.83	1.71 ± 1.02	1.77 ± 0.67	1.98 ± 0.80
NEUT (%)	28.79 ± 9.14	20.62 ± 9.11	24.49 ± 7.14	20.38 ± 6.09	24.32 ± 9.15	20.28 ± 4.98
EO (%)	0.34 ± 0.21	0.36 ± 0.20	0.19 ± 0.13	0.36 ± 0.25	0.46 ± 0.16	0.44 ± 0.28
BASO (%)	0.24 ± 0.10	0.22 ± 0.09	0.20 ± 0.08	0.28 ± 0.18	0.21 ± 0.09	0.27 ± 0.08
LYMPH (10^9^)	9.90 ± 1.58	9.07 ± 1.66	10.92 ± 1.56	9.95 ± 1.87	9.45 ± 1.34	9.55 ± 1.82
MONO (10^9^)	0.21 ± 0.13	0.18 ± 0.13	0.18 ± 0.12	0.24 ± 0.17	0.24 ± 0.13	0.24 ± 0.08
NEUT (10^9^)	4.29 ± 1.90	2.84 ± 1.29	3.66 ± 1.26	2.63 ± 0.77^a^	3.38 ± 2.01	2.48 ± 0.57
EO (10^9^)	0.05 ± 0.02	0.05 ± 0.03	0.03 ± 0.02	0.06 ± 0.04	0.06 ± 0.02	0.06 ± 0.03
BASO (10^9^)	0.03 ± 0.01	0.03 ± 0.01	0.03 ± 0.02	0.04 ± 0.02	0.03 ± 0.01	0.03 ± 0.02
RBC (10^9^)	5.66 ± 0.85	5.78 ± 0.98	6.05 ± 0.57	5.94 ± 0.36	5.93 ± 0.56	5.91 ± 0.70
HGB (g/L)	134.20 ± 19.32	138.10 ± 22.10	139.90 ± 13.35	137.30 ± 7.44	133.80 ± 11.55	134.50 ± 12.70
HCT (%)	35.52 ± 5.02	36.17 ± 6.25	37.88 ± 3.56	37.27 ± 2.30	35.98 ± 3.02	36.31 ± 3.19
MCV (fL)	62.92 ± 2.30	62.59 ± 1.88	62.58 ± 1.07	62.81 ± 1.49	60.74 ± 1.56	61.67 ± 2.57
MCH (pg)	23.76 ± 0.62	23.93 ± 0.55	23.10 ± 0.52	23.14 ± 0.49	22.58 ± 0.47	22.82 ± 0.73
MCHC (g/L)	377.80 ± 8.53	382.80 ± 12.81	369.30 ± 5.62	368.60 ± 5.89	371.70 ± 3.68	370.40 ± 5.66
RDW-SD (fL)	32.34 ± 4.44	31.43 ± 3.52	33.24 ± 3.82	31.37 ± 2.60	30.76 ± 2.34	30.59 ± 1.49
RDW-CV (%)	12.39 ± 1.29	12.07 ± 1.07	12.78 ± 1.39	12.03 ± 0.89	12.20 ± 0.79	11.96 ± 0.33
PLT (10^9^)	979.50 ± 174.99	886.20 ± 181.02	974.90 ± 126.47	904.70 ± 128.98	935.20 ± 69.28	965.90 ± 111.33
MPV (fL)	5.99 ± 0.31	6.26 ± 0.36	6.23 ± 0.20	6.32 ± 0.40	6.02 ± 0.24	6.06 ± 0.15
PCT (%)	0.58 ± 0.10	0.54 ± 0.10	0.61 ± 0.08	0.56 ± 0.08	0.56 ± 0.04	0.59 ± 0.07
PDW (%)	14.89 ± 0.14	14.99 ± 0.18	14.99 ± 0.13	15.01 ± 0.13	14.84 ± 0.11	14.90 ± 0.09

WBC, white blood cell; LYMPH, lymphocyte; MONO, monocyte; NEUT, neutrophil; EO, eosinophil; BASO, basophil; RBC, red blood cell; HGB, hemoglobin; HCT, hematocrit; MCV, mean corpuscular volume; MCH, mean corpuscular hemoglobin; MCHC, mean corpuscular hemoglobin concentration; RDW-SD, red blood cell distribution width-standard deviation; RDW-CV, red blood cell volume distribution width-coefficient of variance; PLT, platelet; MPV, mean platelet volume; PCT, plateletcrit; PDW, platelet distribution width. The values are expressed as the means ± SD of 10 rats in each group. Compared to the control group, a, *p <* 0.05.

#### Micronucleus test of bone marrow cells

There was no obvious poisoning in male and female mice administered different dosages of UW. The micronucleus effect of polychromatic erythrocytes in the bone marrow of male and female mice was not found when UW dose was up to 20,000 mg/kg (Table 4).

**TABLE 4:** Mouse micronucleus test of bone marrow cells.

Group	Dose (mg/kg)	Number of animals ♀/♂	Number of tested PCE ♀/♂	Number of MNPCE ♀/♂	MNCF (‰) ♀/♂	PCE/NCE ♀/♂
Negative control	20 mL/kg	5/5	5,000/5,000	9/10	1.80 ± 0.84/ 2.00 ± 1.00	1.03 ± 0.09/ 1.05 ± 0.08
Positive control	40 mg/kg	5/5	5,000/5,000	114/136	22.80 ± 7.66^b^/ 27.20 ± 5.67^b^	0.98 ± 0.09/ 0.96 ± 0.07
	5,000	5/5	5,000/5,000	11/12	2.20 ± 0.84/ 2.40 ± 1.14	1.06 ± 0.08/ 1.06 ± 0.09
UW	10,000	5/5	5,000/5,000	9/13	1.80 ± 1.10/ 2.60 ± 0.89	1.07 ± 0.02/ 1.08 ± 0.05
	20,000	5/5	5,000/5,000	12/12	2.40 ± 0.89/ 2.40 ± 0.89/	1.04 ± 0.03/ 1.04 ± 0.05

PCE, polychromatic erythrocyte; MNPCE, micronucleated polychromatic erythrocyte; MNCF, micronucleus cell frequency; NCE, normochromic erythrocyte. MNCF (%) and PCE/NCE were counted per mouse. The values are expressed as the means ± SD of five mice in each group. Compared to the negative control group, b, *p <* 0.01.

#### Ames test

As shown in Table 5, the numbers of the revertant colonies in all the UW groups (i.e., low-, medium-, and high-dose groups) were less than twice those of the corresponding solvent control group, regardless of whether S9 was added to the culture system or not. There was also no dose-response relationship. In summary, UW did not induce gene mutations in *S. typhimurium* strains TA97a, TA98, TA100, and TA102.

**TABLE 5:** Ames test result (‾x ± s, *n* = 3).

Group	Dose	TA97a		TA98		TA100		TA102	
		+S_9_	−S_9_	+S_9_	−S_9_	+S_9_	−S_9_	+S_9_	−S_9_
Blank control		120 ± 6	121 ± 6	32 ± 4	33 ± 3	129 ± 5	133 ± 4	306 ± 8	291 ± 14
Solvent control	100 μL/dish	125 ± 9	125 ± 7	32 ± 3	29 ± 2	128 ± 4	125 ± 10	312 ± 10	285 ± 12
UW	12.5 μL/dish	126 ± 5	116 ± 12	31 ± 2	30 ± 2	123 ± 10	129 ± 4	283 ± 13	275 ± 10
UW	25 μL/dish	114 ± 10	120 ± 5	31 ± 2	31 ± 2	131 ± 6	124 ± 1	279 ± 12	271 ± 4
UW	50 μL/dish	118 ± 6	125 ± 7	32 ± l	31 ± 3	129 ± 4	130 ± 11	281 ± 14	281 ± 6
UW	100 μL/dish	123 ± 5	122 ± 1L	31 ± 3	30 ± 2	131 ± 3	130 ± 6	294 ± 7	278 ± 6
Dexon	50 μg/dish		1,050 ± 81		1,136 ± 89				797 ± 62
NaN_3_	1.5 μg/dish						1,151 ± 82		
2-AF	10 μg/dish	953 ± 57		913 ± 75		935 ± 84			
1,8-DHAQ	50 μg/dish							892 ± 38	

Blank control, i.e., the untreated control or the revertant colonies; solvent control, sterile water; 2-AF, 2-aminofluorene; 1,8-DHAQ, 1,8-dihydroxyanthraquinone.

## Discussion

Constipation, characterized by unsatisfactory defecation due to infrequent stools or difficult passage, represents a significant global healthcare burden, with a complex and multifactorial pathophysiology (Scott et al., 2021; Sharma et al., 2021). It is generally believed that increasing water intake helps relieve constipation. However, several studies (Santacruz et al., 2018; Shen et al., 2019; Wegh et al., 2022) have indicated that there is no significant relationship between daily water intake and constipation. Magnesium sulfate-rich mineral water has been used for centuries to treat functional constipation, and its efficacy has been clinically proven (Dupont and Hébert, 2020). Of note, such mineral waters may be inappropriate for patients with cardiovascular diseases (e.g., hypertension) due to their high sodium concentration; moreover, they can easily cause diarrhea if used in large amounts or over the long term. Current management strategies often involve lifestyle modifications and pharmacological interventions, yet the search for safe and effective alternatives continues. Our study provides experimental evidence that UW significantly alleviates constipation in a rat model. The efficacy of UW was demonstrated not only by the reversal of diphenoxylate-induced physical symptoms but also through the normalization of key gastrointestinal hormones, positioning it as a novel non-pharmacological approach.

Constipation is a common clinical condition characterized by reduced defecation frequency and difficulty, which often impairs the patient’s quality of life. In the current study, the SD rats with constipation showed symptoms of poor mental state, clumping, reduced activity, reduced food intake, abdominal distension, hair standing on end and shedding, fewer defecation times and volumes, smaller stool size, bulbous stool, and dry, hard stool. After they were administered TBL suspensions and UW, respectively, the above-mentioned symptoms of the SD rats were improved, for example, fewer defecation times and volume, increased food intake, increased activity, and wetter stool, along with improvement in the appearance, mental state, and diet (Xie and Zhou, 2014). In summary, this study indicated that UW can significantly improve constipation symptoms.

Weight loss is one of the main manifestations of constipation. Adjusting the diet to avoid weight loss is an important way to improve constipation (Feng et al., 2015). In this study, the trend and growth percentage of body weight in SD rats were used to evaluate the effect of UW on improving constipation of the SD rats. It was clearly observed that UW significantly improved weight loss in SD rats and exhibited the same purgative effect as TBL suspension (Figure 2).

Beyond the observed general behavioral improvements and body weight normalization in SD rats, this study specifically evaluated a panel of key gastrointestinal hormones and neurotransmitters (i.e., MTL, SP, VIP, GAS, AChE, ET, and SS) to elucidate the purgative mechanism of UW. Our findings suggest that UW’s laxative effect is mediated through a multi-target modulation of these biomarkers, primarily by enhancing excitatory signals and attenuating inhibitory influences on gut motility.

Our study demonstrated a significant increase in serum MTL levels following UW administration. This finding is consistent with the established literature, which indicates that MTL stimulates phase III of the migrating motor complex, enhances water and electrolyte transport into the gastrointestinal lumen, hydrates fecal matter, and promotes peristalsis to accelerate defecation (Sanger and Furness, 2015). Thus, UW likely facilitates bowel movement by upregulating MTL.

Concurrently, SP levels were significantly elevated in response to UW treatment. SP, a principal excitatory neurotransmitter and tachykinin in the enteric nervous system, promotes motility by directly contracting the longitudinal and circular muscles of the gastrointestinal tract via neurokinin-1 (NK-1) receptors (Sun et al., 2019). This dual action synergistically stimulates propulsive intestinal movement. The observed increase in SP is consistent with recent findings that effective laxative therapies often modulate enteric neurotransmitters to restore motility (Hu et al., 2019). Therefore, we posit that UW exerts its purgative effect by stimulating the release of SP, which, in turn, enhances gastrointestinal contraction and coordinated peristalsis, ultimately accelerating fecal transit.

VIP is another type of neurotransmitter, but the relationship between the *in vivo* level of VIP and constipation is controversial. In theory, as an inhibitory neurotransmitter, elevated VIP can inhibit gastrointestinal motility and lead to constipation, but a few animal experiments found that the serum VIP level increased after constipation symptoms were improved (Huang et al., 2016), which was consistent with our result. Changes in VIP levels, whether increases or decreases, are closely associated with the development of constipation. Maintaining the normal levels of VIP in the intestinal wall is an important means of stabilizing gut function (Zhao et al., 2018). Therefore, it may be deduced that UW improves constipation symptoms, possibly by maintaining normal levels of VIP.

Contrary to some expectations, serum GAS levels were not significantly altered by UW treatment. As GAS primarily stimulates gastric acid secretion and influences gastric emptying, its unaltered state suggests that the purgative effect of UW is not mediated through the gastrin-dependent pathway (Tan et al., 2021).

The cholinergic system plays a fundamental role in initiating gut peristalsis. AChE, the enzyme responsible for hydrolyzing acetylcholine (ACh), serves as a reliable marker for cholinergic activity. An increase in AChE activity typically reflects an enhanced release of its substrate, ACh (Qiu et al., 2007). Our data indicated a significant increase in AChE levels after UW administration. This leads us to conclude that UW promotes intestinal contraction and mucous secretion by augmenting cholinergic signaling via increased ACh release, thereby propelling stool forward (Wang et al., 2015).

ET is an important factor in regulating cardiovascular function and plays a key role in maintaining vascular tension and cardiovascular system (Qian et al., 2013). An increase in ET promotes normal contraction and diastole of blood vessels and relieves abnormal contractions caused by constipation (Chen et al., 2019). In our study, the level of serum ET in the UW group showed no difference compared with the other groups, indicating that the purgative mechanism of UW is independent of alterations in systemic ET levels.

Different from the above-mentioned hormones, SS is the only inhibitory hormone among the seven hormones that can inhibit the release of other excitatory gastrointestinal hormones. The significant decrease in serum SS levels following UW treatment is particularly noteworthy. By reducing SS-mediated inhibition, UW likely disinhibits gastric emptying and smooth muscle contraction, thereby creating a net excitatory environment conducive to alleviating constipation (Tan et al., 2021).

**FIGURE 7:**
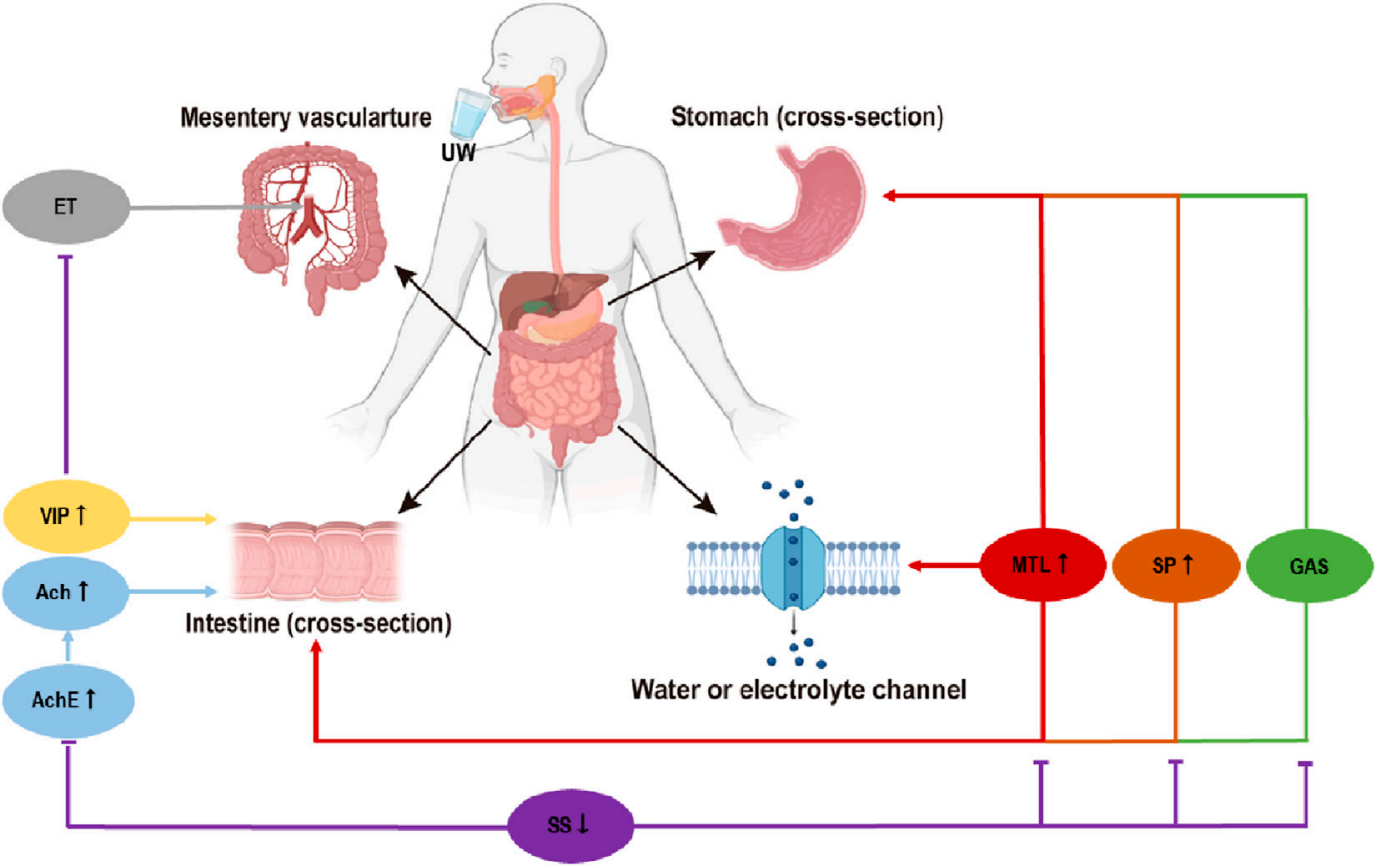
Purgative mechanisms of UW. Note: UW, ultrasonic water; ET, endothelin; VIP, vasoactive intestinal peptide; ACh, acetylcholine; AChE, acetylcholinesterase; SS, somatostatin; MTL, motilin; SP, substance P; GAS, gastrin.

In summary, UW exerts its comprehensive purgative effect through a coordinated modulation of neuro-hormonal pathways. It enhances pro-kinetic signals by elevating MTL, SP, and AChE activities while concurrently reducing the inhibitory brake on gut motility by lowering SS levels. The effect on VIP appears to be one of normalization rather than simple upregulation or downregulation. This multifaceted action, graphically summarized in Figure 7, underscores UW’s potential as an effective agent for managing constipation by restoring the dynamic balance of gastrointestinal regulators.

In addition to the regulation of gastrointestinal hormones, the purgative effect of UW may also be related to its unique physical structure. The cluster network structure of UW [n × (H_2_O)_n_], as supported by spectroscopic evidence, could potentially enhance the absorption and transport of substances in the gastrointestinal tract (Xia, 2019). Dietary fiber is fermented by bacteria in the colon to produce hydrogen, which is then utilized by methanogenic bacteria in the colon to generate methane (Sharma and Rao, 2017). A study found that the infusion of methane gas resulted in a delay of gastrointestinal transit and reduced contractility (Pimentel et al., 2006), and both pediatric (Soares et al., 2005) and adult (Attaluri et al., 2010) slow transit constipation (STC) patients had an increased prevalence of methanogenic flora. In other words, UW could facilitate the absorption of methane gas produced by methanogenic flora through its cluster network structure n × (H_2_O)_n_, thereby promoting gastrointestinal transit and contractility.

Safety evaluation is an essential step in the development and application of a new product. As a new type of functional drinking water, the safety of UW should not be ignored. Predictably, UW exhibits reliable security since it is processed via physical ways (i.e., ultrasonic purification technology). UW is safer and healthier than ordinary drinking water because the by-products of the water, such as trichloromethane and tetrachloromethane, have been effectively removed using ultrasonic water equipment (Xia, 2019). However, the safety of UW should be further verified and systematically evaluated through a series of safety tests, including an acute oral toxicity test, a subacute oral toxicity test, a micronucleus test of bone marrow cells, and an Ames test.

The results of the acute oral toxicity test showed that the male and female mice administered with 20,000 mg/kg of UW exhibited no mortality or obvious signs of poisoning within 2 weeks. It indicated that UW was practically non-toxic. In the subacute oral toxicity test, general behavior, serum biochemical indices, blood routine indices, and the histopathological examination of the SD rats were observed and evaluated. The results showed that nearly all the evaluation indices of the SD rats show no statistically significant differences between the UW group and the control group (the purified water group). Although a few serum biochemical indexes (i.e., UA, AST, and total protein) and blood routine indices (i.e., NEUT) in the rats showed a statistically significant difference between the UW and control groups, these differences were not clinically significant since all values remained within the normal physiological range. The histopathological experiment results also indicated that all the organs and tissues of the male and female rats showed no significant pathological changes or toxicological characteristics.

As a rapid and effective *in vitro* test method, the micronucleus test of bone marrow cells was used to evaluate genotoxicity in the present study. It is an *in vitro* cytogenetic test that uses erythrocytes in the bone marrow of rodents to detect chemical damage to the chromosomes or mitotic apparatus of mammalian cells. During erythroblast development into PCEs in the bone marrow, the main nucleus is extruded, so any micronucleus (MN) that has been formed may remain in the otherwise enucleated cytoplasm. The damage in the chromosome appears as a small additional nucleus and is readily identifiable under a light microscope. An increase in the frequency of micronucleated polychromatic erythrocytes (MN PCEs) in treated animals is an indication of genotoxicity (Jain and Pandey, 2019). The results of the micronucleus test indicated that UW had no micronucleus effect on bone marrow cells of the mice when the dose was up to 20,000 mg/kg.

The Ames test provides a rapid and relatively simple procedure for evaluating mutagenicity and DNA damage of chemicals, which is widely used to screen for potential germ cell mutagens and carcinogens rapidly and inexpensively (Zeiger, 2019). The results of the Ames test are usually determined by calculating the number of revertant colonies growing on the culture medium. If the number of revertant colonies in the test substance group (e.g., the UW group) is twice or more than that in the solvent control group and there is a dose–response relationship or at least one test point shows a repeatable and statistically significant positive reaction, the test substance mutation test for the substance can be considered positive. After the test substance is determined using the four test strains (i.e., *S. typhimurium* strains TA97a, TA98, TA100, and TA102), and the result of one test strain is positive, regardless of whether it is in the absence of S9 or in the presence of S9, it can be determined that the test substance is mutagenic positive to *S. typhimurium* strains. On the contrary, as long as the results of all four test strains are negative, whether in the absence or presence of S9, it can be determined that the test substance is non-mutagenic. In the present study, the numbers of revertant colonies in all UW groups, with or without S9, were not more than two-fold higher than those in the solvent control group, and there was no dose-response relationship. Therefore, it can be concluded that UW is non-mutagenic.

It is important to note that although the laxative effect and safety of UW have been preliminarily confirmed through our *in vivo* and *in vitro* experiments, this research was conducted in a rodent model. Such models may not completely recapitulate the complex pathophysiology of human constipation. Therefore, the findings cannot be directly translated to human applications. Clinical trials are warranted to validate the efficacy and safety of UW in humans. Furthermore, a chronic toxicity study (over 90 days) is an essential next step to fully ascertain its safety for long-term human consumption. The underlying molecular mechanisms also require further elucidation through advanced techniques such as molecular biology (e.g., Western blot and RT-qPCR) and multi-omics analyses (e.g., metagenomics, transcriptomics, proteomics, and metabolomics).

## Conclusion

As a new type of functional drinking water, UW exhibited a favorable purgative effect by improving general behavior, body weight, and serum biochemical indices in SD rats with constipation. The purgative mechanisms of UW were mainly attributed to its regulation of serum biochemical indices related to constipation, i.e., increasing serum levels of excitatory neurotransmitters (i.e., MTL, SP, VIP, and AChE) and decreasing serum levels of inhibitory neurotransmitters (i.e., SS), which promoted gastro-intestinal peristalsis and increased gastric evacuation. The safety of UW was evaluated through an acute oral toxicity test, a subacute oral toxicity test, a micronucleus test of bone marrow cells, and an Ames test, which together served as the basis for permitting UW as a functional drinking water. All the results of safety evaluation, both *in vivo* and *in vitro*, showed that UW was safe and non-toxic.

## Data Availability

The original contributions presented in the study are included in the article/Supplementary Material; further inquiries can be directed to the corresponding authors.

## Glossary

UW: Ultrasonic water
MTL: Motilin
GAS: Gastrin
ET: Endothelin
SS: Somatostatin
AChE: Acetylcholinesterase
SP: Substance P
VIP: Vasoactive intestinal peptide
WBC: White blood cell
RBC: Red blood cell
ALT: Alanine aminotransferase
AST: Aspartate aminotransferase
TBIL: Total bilirubin
BUN: Urea nitrogen
GLO: Globulin
TG: Triacylglycerol
GLU: Glucose
TP: Total protein
ALB: Albumin
CRE: Creatinine
TBL: Tongbianling
UA: Uric acid
CHO: Cholesterol
LYMPH: Lymphocyte
MONO: Monocyte
NEUT: Neutrophil
EO: Eosinophil
BASO: Basophil
HGB: Hemoglobin
HCT: Hematocrit
MCV: Mean corpuscular volume
MCH: Mean corpuscular hemoglobin
MCHC: Mean corpuscular hemoglobin concentration
RDW-SD: Red blood cell distribution width-standard deviation
RDW-CV: Red blood cell volume distribution width-coefficient of variance
PLT: Platelet
MPV: Mean platelet volume
PCT: Plateletcrit
PDW: Platelet distribution width
PCE: Polychromatic erythrocyte
MNPCE: Micronucleated polychromatic erythrocyte
MNCF: Micronucleus cell frequency
NCE: Normochromic erythrocyte
