# Supplementary material for: A new type of ultrasonic water alleviates constipation with favorable safety
Source: Front Toxicol. 2025 Nov 20;7:1679872. doi: 10.3389/ftox.2025.1679872 (PMC12675270; doi:10.3389/ftox.2025.1679872)
Supplement: Supplementary file 1 [file Table1.docx]

**Supplementary Table 1** **The serum biochemical indexes of the rats in the normal, model, positive and UW group (n = 8).**

|  | | Normal group | Model group | Positive group | UW group |
| --- | --- | --- | --- | --- | --- |
| MTL  (pg/ml) | I | 1239.63±116.47 a | 1274.13±153.64 a | 1225.13±72.00 a | 1267.42±189.59 a |
|  | II | 1207.27±104.41 a | 921.41±52.71 c | 982.99±80.77 c | 966.95±42.41 c |
|  | III | 1212.70±42.79 a | 1151.85±91.95 b | 1213.39±22.49 a | 1200.18±46.37 a |
| SP | I | 260.25±37.23 a | 256.98±26.14 ab | 264.42±24.38 a | 255.83±28.46 a |
| (ng/L) | II | 266.17±23.51 a | 119.86±21.42 c | 121.01±13.21 c | 109.36±14.17 c |
|  | III | 271.90±28.99 a | 242.67±32.33 b | 273.21±4.32 a | 269.05±12.02 a |
| VIP  (ng/L) | I | 210.07±22.51 a | 208.00±7.23 a | 210.60±13.31 a | 209.94±11.91 a |
|  | II | 212.60±6.13 a | 117.32±12.44 d | 125.77±18.46 cd | 139.54±15.78 c |
|  | III | 212.47±11.77 a | 152.84±15.44 b | 211.39±9.61 a | 208.91±12.18 a |
| GAS  (ng/L) | I | 92.39±7.81 a | 92.39±9.85 a | 97.08±9.50 a | 96.01±4.01 a |
|  | II | 90.54±2.37 a | 76.88±9.07 b | 66.24±5.46 c | 66.28±7.46 c |
|  | III | 91.56±7.52 a | 86.72±11.72 ab | 90.92±5.93 a | 91.46±3.3 a |
| AchE  (nmol/L) | I | 304.22±33.34 a | 308.93±17.60 a | 302.38±35.66 a | 296.54±23.86 a |
|  | II | 299.51±22.69 a | 256.58±13.67 bc | 254.36±11.47 c | 253.28±12.99 c |
|  | III | 300.21±30.88 a | 275.38±22.73 b | 308.71±26.40 a | 310.88±10.51 a |
| ET  (µg/L) | I | 153.51±8.24 a | 150.85±12.11 a | 158.27±7.66 a | 150.26±10.77 a |
|  | II | 148.29±3.04 a | 80.15±4.85 b | 73.58±10.10 b | 75.60±8.82 b |
|  | III | 150.42±17.26 a | 138.29±12.43 a | 150.85±12.65 a | 152.55±15.13 a |
| SS  (ng/L) | I | 118.03±8.72 c | 119.21±10.94 bc | 122.47±9.14 c | 117.19±11.66 c |
|  | II | 112.30±8.36 c | 505.60±76.08 a | 548.55±64.67 a | 501.09±36.19 a |
|  | III | 119.27±7.58 c | 160.03±7.95 b | 122.43±9.34 c | 124.19±6.17 c |

Note: I, before the compound diphenoxylate suspension were given the rats (Day 0); II, when the rat constipation models were successfully established (Day 9); III, at the end of the whole experiment (Day 23). Normal group represents the normal SD rats; Model group represents the SD rats with constipation; Positive group represents the positive control SD rats administrated TBL suspension (250 mg/kg); UW group represents the SD rats given ultrasonic water. MTL, motilin; SP, substance P; VIP, vasoactive intestinal peptide; GAS, gastrin; AchE, acetylcholinesterase; ET, endothelin; SS, somatostatin. a, b, c, the mean values with different letters over the bars are significantly different (*P* < 0.05), the mean values with the same letters over the bars showed no significantly different (*P* ＞ 0.05).

**Supplementary Table 2** **Histopathological characteristics of organs and tissues of the SD rats in the control and UW group.**

| **Organs and Tissues** | **Histopathological Characteristics** |
| --- | --- |
| Brain | Tissues of the cerebrum and cerebellum were visible. The cortex and medulla structure were clear, and neurons were scattered. |
| Heart | The myocardial fibers were clearly striated, the nucleus was centered, and there were a few blood vessels and connective tissue in the mesenchyme. |
| Liver | Structures of the hepatic lobule were normal, and the blood sinuses were clear. Some small bile ducts were dilated with cholestasis. |
| Spleen | Structures of the red pulp and white pulp were clear. There was mild congestion in the focal area. |
| Lung | The pulmonary alveoli were filled and normal. The pulmonary septum in the focal area was widened. Small blood vessels were dilated. A small number of inflammatory cells were infiltrated in the focal area. Bronchi were covered by the ciliated columnar epithelium. |
| Kidney | Proportion of the cortex to the medulla was normal. Structures of the glomerulus and renal tubules were clear. The spontaneous calcified bodies were visible in the focal area. The renal pelvis was covered by the normal urothelium. |
| Stomach | The layers of gastric wall were clear. The fundus gland and pyloric gland were well developed. The gastric pits were visible. The main cells and parietal cells were orderly distributed. The stomach surface was covered by the squamous epithelium with keratosis. |
| Intestines | The layers of intestinal wall are clear. Structures of the lobular and columnar villi were clear. Distribution of the goblet cells and Pan's cells was normal. The lymphoplasmic cells were clustered in the lamina propria of mucosa. The lymphoid tissue in the submucosa proliferated, and there was lymphoid follicular formation. |
| Uterus | The endometrium was normal, and was covered with single layer of columnar epithelium. |
| Ovary | Structures of the cortex and medulla were normal. Follicles at all levels were visible in the cortex. No luteum. |
| Testis | Structure of the testis was normal. The spermatocytes at all levels were arrange in layers in the seminiferous tubules. The free sperm cells were visible in the central lumen. |
| Skeletal Muscle | The transverse section of muscle tissue showed that, there were several muscle fiber bundles with clear boundaries. The muscle fibers were closely arranged. Appearance of most muscle fibers is angular, and occasionally circular. There were not degenerative and necrotic muscle fibers as well as abnormal substances precipitation. There was not obvious inflammatory cell infiltration around the blood vessels. |
